# Supplementary material for: Whole-Exome Sequencing Reveals High Mutational Concordance between Primary and Matched Recurrent Triple-Negative Breast Cancers
Source: Genes (Basel). 2023 Aug 25;14(9):1690. doi: 10.3390/genes14091690 (PMC10531222; doi:10.3390/genes14091690)
Supplement: Supplementary file 1 [file genes-14-01690-s001.zip › genes-2522916-supplementary.pdf]

Supplementary Table S1: DNA amount and DNA integrity number for primary tumors that recurred (P; n=11); recurrent tumors (R; n=9) and primary tumors that did not recur (P'; n=15)

**Sample**	**Amount (µg)**	**DNA Integrity Number**
P1	0.31	5.4
R1	1.10	5.6
P2	0.18	4.7
R2	0.55	4.5
P3	0.09	3.2
R3	0.14	4.2
P4	0.88	4.4
R4	0.06	4.9
P5	0.11	4.7
R5	0.13	4.5
P6	0.50	4.4
R6	0.18	4.4
P7	0.22	4.4
R7	0.49	4.9
P8	0.06	3.4
R8	0.13	4.9
P9	0.19	4.1
R9	0.02	2.7
P10	0.06	3.5
P11	0.12	4.1
P1'	0.24	3.5
P2'	1.15	5.4
P3'	0.48	4.0
P4'	3.24	4.8
P5'	0.25	5.0
P6'	1.40	4.4
P7'	0.22	3.5
P8'	0.41	4.4
P9'	0.88	4.1
P10'	0.31	4.0
P11'	1.58	4.8
P12'	0.18	4.2
P13'	0.48	4.0
P14'	0.10	3.5
P15'	1.35	4.6

Supplementary Table S2. Summary of SNVs and indels in paired primary (P; n=9) and recurrent (R; n=9) tumors

			Frameshift deletion	Frameshift insertion	In-frame deletion	In-frame insertion	Missense	Nonsense	Non-stop	Splice-site	Translation start site
**P1**	SNV	312					254	25	2	27	4
	Indel	221	81	23	64	53					
**R1**	SNV	336					242	40	0	51	3
	Indel	259	102	31	65	61					
**P2**	SNV	319					242	48	2	24	3
	Indel	257	101	28	61	67					
**R2**	SNV	705					459	117	8	113	8
	Indel	645	371	69	135	70					
**P3**	SNV	431					296	57	4	72	2
	Indel	301	145	31	68	57					
**R3**	SNV	456					309	73	5	65	4
	Indel	325	165	45	62	53					
**P4**	SNV	405					288	51	7	56	3
	Indel	319	144	39	74	62					
**R4**	SNV	301					218	44	3	33	3
	Indel	228	69	26	73	60					
**P5**	SNV	303					226	34	1	37	5
	Indel	219	83	24	57	55					
**R5**	SNV	334					247	42	3	36	6
	Indel	237	88	27	63	59					
**P6**	SNV	458					331	59	3	61	4
	Indel	306	149	28	63	66					
**R6**	SNV	573					390	90	6	65	7
	Indel	415	200	49	93	73					
**P7**	SNV	431					305	58	1	63	4
	Indel	362	169	46	71	76					
**R7**	SNV	509					362	66	9	65	7
	Indel	360	156	39	94	71					
**P8**	SNV	514					342	80	4	86	2
	Indel	445	255	39	85	66					
**R8**	SNV	380					263	65	1	48	3
	Indel	278	97	35	81	65					
**P9**	SNV	535					389	82	5	57	2
	Indel	354	173	40	85	56					
**R9**	SNV	2179					1552	358	12	249	8
	Indel	2089	1430	343	220	96					

Supplementary Table S3. Summary of SNVs and indels in primary tumors that recurred (P; n=11) and primary tumors that remained recurrence-free (P'; n=15)

			Frameshift deletions	Frameshift insertion	In-frame deletion	In-frame insertion	Misse nse	Nons ense	Non-stop	Splice-site	Translation start site
**P1**	SNV	312					254	25	2	27	4
	Indel	221	81	23	64	53					
**P2**	SNV	319					242	48	2	24	3
	Indel	257	101	28	61	67					
**P3**	SNV	431					296	57	4	72	2
	Indel	301	145	31	68	57					
**P4**	SNV	405					288	51	7	56	3
	Indel	319	144	39	74	62					
**P5**	SNV	303					226	34	1	37	5
	Indel	219	83	24	57	55					
**P6**	SNV	458					331	59	3	61	4
	Indel	306	149	28	63	66					
**P7**	SNV	431					305	58	1	63	4
	Indel	362	169	46	71	76					
**P8**	SNV	514					342	80	4	86	2
	Indel	445	255	39	85	66					
**P9**	SNV	535					389	82	5	57	2
	Indel	354	173	40	85	56					
**P10**	SNV	2647					2106	314	10	203	14
	Indel	1171	774	72	294	31					
**P11**	SNV	1138					864	156	7	105	6
	Indel	579	365	56	119	39					
**P1'**	SNV	396					286	52	7	46	5
	Indel	272	98	37	84	53					
**P2'**	SNV	279					219	23	1	34	2
	Indel	208	72	18	53	65					
**P3'**	SNV	435					343	61	4	82	5
	Indel	389	197	36	93	63					
**P4'**	SNV	321					229	42	1	45	4
	Indel	240	97	22	59	62					
**P5'**	SNV	382					282	53	4	39	4
	Indel	266	111	35	62	58					
**P6'**	SNV	593					443	78	6	63	3
	Indel	346	184	39	75	48					
**P7'**	SNV	398					278	52	5	59	4
	Indel	297	111	36	81	69					
**P8'**	SNV	416					284	54	4	69	5
	Indel	280	107	33	80	60					
**P9'**	SNV	535					372	74	8	77	4
	Indel	354	180	30	86	58					
**P10'**	SNV	364					246	60	2	54	2

	Indel	289	115	33	77	64					
**P11'**	SNV	440					317	60	2	55	6
	Indel	317	144	34	79	60					
**P12'**	SNV	352					278	36	6	31	1
	Indel	263	99	29	69	66					
**P13'**	SNV	390					288	47	4	47	4
	Indel	317	121	41	88	67					
**P14'**	SNV	820					634	93	1	84	8
	Indel	369	210	33	84	42					
**P15'**	SNV	535					413	63	1	56	2
	Indel	294	123	30	78	63					

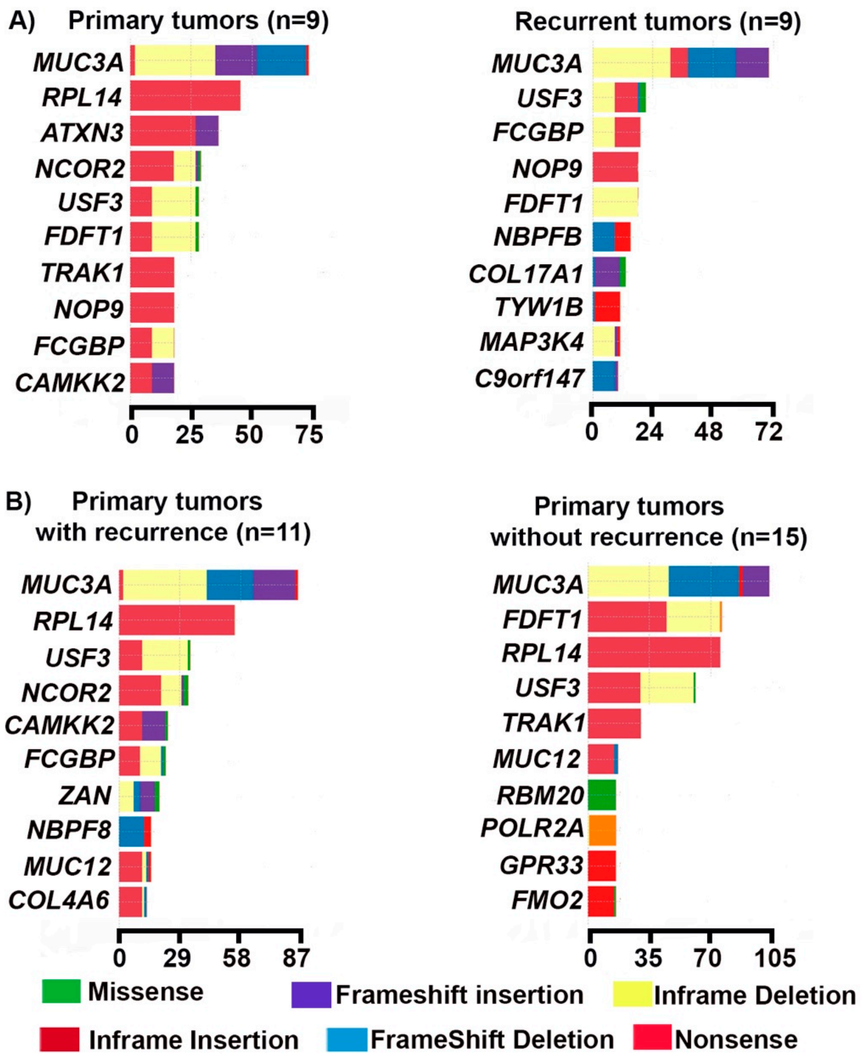

Supplementary Figure S1. MAF plot showing the top 10 most commonly mutated genes in primary **(A)** and matched recurrent tumors (n=9) and **(B)** in primary tumors with (n=11) and without recurrence (n=15).
